# Risk for Mycobacterial Disease among Patients with Rheumatoid Arthritis, Taiwan, 2001–2011

**DOI:** 10.3201/eid2108.141846

**Published:** 2015-08

**Authors:** Tsai-Ling Liao, Ching-Heng Lin, Gwan-Han Shen, Chia-Li Chang, Chin-Fu Lin, Der-Yuan Chen

**Affiliations:** Taichung Veterans General Hospital, Taichung, Taiwan (T.-L. Liao, C.-H. Lin, G.-H. Shen, C.-L. Chang, C.-F. Lin, D.-Y. Chen);; National Chung Hsing University, Taichung (T.-L. Liao, G.-H. Shen, D.-Y. Chen);; National Taipei University of Nursing and Health Science, Taipei, Taiwan (C.-H. Lin);; National Yang Ming University, Taipei (D.-Y. Chen);; Chung Shan Medical University, Taichung (D.-Y. Chen)

**Keywords:** tuberculosis, nontuberculous mycobacterial disease, risk factor, rheumatoid arthritis, death hazard, tuberculosis and other mycobacteria, Taiwan

## Abstract

Increasing evidence indicates that the risk of acquiring tuberculosis (TB) and nontuberculous mycobacterial disease is elevated among patients with rheumatoid arthritis (RA). To determine the epidemiology of mycobacterial diseases among RA patients in Asia, we conducted a retrospective cohort study. We used a nationwide database to investigate the association of RA with mycobacterial diseases. The risk for development of TB or nontuberculous mycobacterial disease was 2.28-fold and 6.24-fold higher among RA patients than among the general population, respectively. Among RA patients, risk for development of mycobacterial disease was higher among those who were older, male, or both. Furthermore, among RA patients with mycobacterial infections, the risk for death was increased. Therefore, RA patients, especially those with other risk factors, should be closely monitored for development of mycobacterial disease.

## Abstract 2

Risk for illness and death are increased for these patients.

The major clinical spectrum of mycobacterial diseases is caused by tuberculosis (TB) and nontuberculous mycobacteria (NTM). TB remains a major global health problem; in 2012, an estimated 8.6 million persons became infected and 1.3 million died of the disease (*1*). NTM are ubiquitous environmental microorganisms that cause chronic pulmonary and extrapulmonary infection in patients with inflammatory diseases (*2*). Several NTM strains are resistant to many antimicrobial drugs, making treatment difficult (*3*). Because reporting of NTM disease to public health administrations is not required in most countries, epidemiologic data for these countries are not available (*4*). Pulmonary diseases caused by NTM are being diagnosed with increasing frequency worldwide (*5*), including in Taiwan (*6*). In Taiwan, the incidence of TB remains high, despite extensive implementation of well-known TB control measures and use of the Bacillus Calmette-Guérin vaccine (*7*); between 2000 and 2012, a laboratory-based study indicated a trend of decreasing TB cases but significantly increasing NTM cases in Taiwan (*6*). 

Rheumatoid arthritis (RA), a chronic articular inflammatory disease (*8*), affects 0.5%–1.0% of the adult population and is a major cause of disability in industrialized countries (*9*,*10*). Among RA patients, the risks of acquiring or dying of an infectious disease are increased, possibly because of disease-related immune dysfunction or the immunosuppressive effects of therapeutic agents (*11*). In Europe and the United States, an increased risk for TB among RA patients has been reported (*12*), and the risk for active TB is even higher among those receiving anti–tumor necrosis factor α (TNF-α) therapy (*13*). Previous clinical studies have shown that the prevalence of latent tuberculosis infection was higher among RA patients than among healthy controls (*14*). A recent study indicated that in the United States, the incidence of NTM disease was significantly higher among RA patients receiving anti–TNF-α therapy than among patients with other inflammatory diseases who were receiving the same treatment (*15*). The prevalence of mycobacterial diseases is higher among the general population in Asia than in the United States and Europe (*1*,*16*). However, few population-based epidemiologic studies have investigated the association of RA with mycobacterial diseases in Asia. In addition, prevalence of concurrent medical conditions is higher among RA patients (*17*,*18*), which may affect their risk for TB (*19*). However, the association of RA with concurrent medical conditions and mycobacterial infection is unclear.

In Taiwan, the National Health Insurance program is a mandatory universal health insurance program that provides comprehensive medical care for >99% of Taiwan’s residents (*20*–*22*). The National Health Insurance Research Database (NHIRD) is managed by the National Health Research Institutes, and confidentiality is maintained according to Bureau of National Health Insurance guidelines (*23*). We used this nationwide database to conduct a retrospective cohort study investigating the association between RA and mycobacterial diseases in Taiwan during 2001–2011.

## Methods

### Data Source

**Figure 1:**
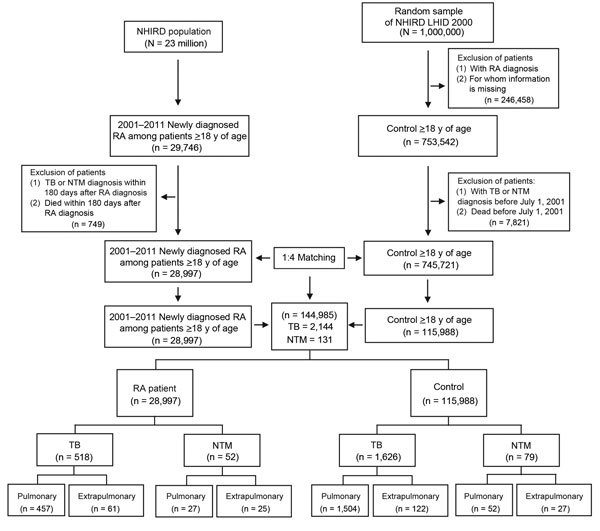
Flowchart of case selection for rheumatoid arthritis (RA) patients and age- and sex-matched controls (without RA) from the National Health Insurance Research Database (NHIRD). LHID 2000, Longitudinal Health Insurance Database 2000; NTM, nontuberculous mycobacteria; TB, tuberculosis.

The NHIRD includes inpatient and ambulatory care claims covering 1996–2011. The Longitudinal Health Insurance Database 2000 contains all original claims data for 1 million persons randomly sampled from the Registry for Beneficiaries of the NHIRD, which was released by the National Health Research Institutes, which confirmed that the random samples were representative of the general population in Taiwan. We systemically sampled NHIRD patient data for 2001–2011 (Figure 1). The data were de-identified forms of secondary information in an anonymous format released to the public for research purposes. All work was done at the Taichung Veterans General Hospital, Taichung, Taiwan, and the institutional review board of this hospital exempted this study from full review (no. CE13151-1).

### Definitions

Patients with different diseases were identified by use of codes from the International Classification of Diseases, Ninth Revision, Clinical Modification (ICD-9-CM). In addition, the NHIRD includes a registry system for catastrophic illnesses, and RA is included in this database. Therefore, the diagnosis of RA (ICD-9-CM code 714.0) was made according to the 1987 American College of Rheumatology criteria (*24*) and the NHIRD Registry of Catastrophic Illness Patient Database.

All TB and NTM cases were determined by use of ICD-9-CM codes, laboratory mycobacterium examination codes, and antimycobacterial therapy receipts; all 3 criteria needed to be met. We used the definition of TB in the published literature (*20*,*21*). First, the ICD-9-CM codes for TB were 010–018. Among TB cases, ICD-9-CM codes 010–012 and 018 are used for pulmonary TB and codes 013–017 are used for extrapulmonary TB. Second, >2 mycobacteria laboratory examination codes were used for TB identification, including those identified by acid-fast smear (13006C/13025C), mycobacterial culture (13012C/13026C), acid-fast bacillus differentiation (13013C), tuberculin test (12106C), tuberculosis test (13024C), and bronchoscopy (28006C) results (*20*). Third, we identified patients who had taken at least 1 prescription consisting of >3 anti-TB drugs used simultaneously for at least 120 days during a 180-day period; these drugs were isoniazid, ethambutol, rifampin, pyrazinamide, amikacin, kanamycin, streptomycin, ciprofloxacin, ofloxacin, moxifloxacin, levofloxacin, prothionamide, clarithromycin, and thioridazine (*20*,*21*).

The NTM disease definition was based on the American Thoracic Society and the Infectious Diseases Society of America guidelines (*3*), which include clinical, radiographic, and microbiologic criteria. All items carry equal weight and all must be met for a diagnosis of NTM lung disease (*3*) as follows. First, the patient must have been assigned an ICD-9-CM code for NTM disease (031.0, 031.1, 031.2, 031.8, and 031.9). Among NTM diseases, ICD-9-CM code 031.0 is for pulmonary NTM disease and the others are for extrapulmonary NTM disease. Second, the mycobacteria laboratory examination code criteria for NTM needed to be consistent with those for TB (*20*). Third, the patient should have received >2 drugs to treat NTM disease, including amikacin, cefoxitin, ciprofloxacin, clarithromycin, doxycycline, ethambutol, imipenem, levofloxacin, meropenem, minocycline, moxifloxacin, rifabutin, rifampin, tigecycline, and streptomycin (*3*). Simultaneous TB and NTM disease was defined as TB only. In addition, the first diagnosis of TB or NTM disease must have been made >6 months after the identification of the RA. The study end point was defined as the onset of new mycobacterial disease or death during the 11-year follow-up period (2001–2011).

To understand the association between RA-related concurrent conditions and mycobacterial diseases, we choose several RA-related conditions to study. The definitions of each condition were based on the following ICD-9-CM codes: cardiovascular diseases (codes 390–438), diabetes mellitus (code 250), hyperlipidemia (code 272), liver cirrhosis (code 571), and chronic obstructive pulmonary disease (codes 490–492 and 496). In addition, we also selected mycobacterial disease-related conditions (e.g., chronic kidney disease [code 585] and HIV disease [codes 042–044]).

### Study Population

The study was a retrospective cohort study of NHIRD data for 29,746 patients >18 years of age who had received a new diagnosis of RA during January 1, 2001, through December 31, 2011. We excluded 749 patients whose TB or NTM diagnosis was made within 180 days after the RA diagnosis and/or who died within 180 days after RA diagnosis. The age-matched control group (>18 years of age) was selected from the NHIRD Longitudinal Health Insurance Database 2000 and excluded patients for whom information regarding age or sex was missing, those with a history of RA, TB, or NTM, and those who died before July 1, 2001. Patients with newly diagnosed RA (28,997 patients) and controls who did not have RA (115,988 controls) were matched 1:4 by age and sex (Figure 1).

### Statistical Analyses

Data are presented as mean ± SD for continuous variables and as proportions for categorical variables. Differences were analyzed by using the independent *t*-test for the continuous variables and the χ^2^ test for the categorical variables. Incidence rates for newly diagnosed mycobacterial diseases among RA patients and controls were calculated. For each cohort, incidence rates per 1,000 person-years were also calculated according to the distribution of demographic characteristics and concurrent conditions. The multivariable Cox proportional hazards model was adjusted for age, sex, Charlson Comorbidity Index score (*25*), and urbanization (residence in urban, suburban, or rural area) before being used to identify independent factors contributing to the development of mycobacterial diseases in the RA and control cohorts. The 95% CI for each variable was also determined. All analyses were conducted by using SAS statistical software version 9.3 (SAS Institute, Inc., Cary, NC, USA). A p value of <0.05 was considered significant.

## Results

### Characteristics of the Study Cohort

Data for ≈99% of Taiwan residents were included in the NHIRD. Among these, 28,997 eligible persons with newly diagnosed RA were identified during 2001–2011. The follow-up period for this study population, during which risk for mycobacterial disease development was assessed; mean follow-up time was 5.1 years (range 2.1–8.3 years). The control group was matched for age and sex at a ratio of 1:4, and a total of 115,988 participants who did not have RA (controls) were identified (Figure 1). Characteristics of the enrolled participants are summarized in Table 1. RA patients were predominately (77.7%) female, and mean age was 53.9 years. Approximately 22.9% of the RA patients were >65 years of age. Age and sex distributions between the RA patients and controls did not differ significantly. Prevalence of concurrent conditions was higher among RA patients than among controls (56.4% vs. 32.1%, respectively; p<0.0001). No clinically significant difference in urbanization was found between RA patients and controls.

**Table 1:** Baseline characteristics for 144,985 patients with and without RA, Taiwan, 2001–2011*

Variables	With RA, no. (%), n = 28,997†	Without RA, no. (%), n = 115,988†	p value
Age at entry, y‡			
18–44	7,495 (25.9)	29,980 (25.9)	1.000
45–64	14,865 (51.3)	59,460 (51.3)	
>65	6,637 (22.9)	26,548 (22.9)	
Sex			
F	22,524 (77.7)	90,096 (77.7)	1.000
M	6,473 (22.3)	25,892 22.3)	
Concurrent conditions			
Cardiovascular disease	8,100 (27.9)	28,327 (24.4)	<0.0001
Diabetes mellitus	2,480 (8.6)	9,749 (8.4)	0.419
Hyperlipidemia	2,951 (10.2)	8,301 (7.2)	<0.0001
Liver cirrhosis	2,451 (8.5)	7,277 (6.3)	<0.0001
COPD	2,097 (7.2)	8,247 (7.1)	0.472
Chronic kidney disease	270 (0.9)	896 (0.8)	0.007
HIV disease	1 (0.003)	9 (0.01)	0.698
Charlson Comorbidity Index score§			
0	12,653 (43.6)	78,773 (67.9)	<0.0001
1	8,708 (30.0)	20,791 (17.9)	
2	4,381 (15.1)	8,823 (7.6)	
>3	3,255 (11.2)	7,601 (6.6)	
Urbanization			
Urban	17,167 (59.2)	68,429 (59.0)	0.668
Suburban	4,098 (14.1)	16,622 (14.3)	
Rural	7,732 (26.7)	30,937 (26.7)	

*COPD, chronic obstructive pulmonary disease; RA, rheumatoid arthritis. †Median, mean follow-up time for patients with RA = 5.1, 5.2 ± 3.1 y and for patients without RA = 11.0, 10.3 ± 2.1 y; p<0.0001. **‡**Mean (± SD) age for patients with RA = 53.9 ± 14.1 y and for patients without RA = 53.5 ± 14.9 y; p<0.0001. §**Mean** (± SD) index for patients with RA = 1.0 ± 1.3 and for patients without RA = 0.6 ± 1.2; p<0.0001.

### Characteristics of RA Patients with Mycobacterial Disease

Among the total study population of 144,985, new-onset TB developed in 2,144 (1.48%) (Figure 1). Among the 28,997 RA patients, 518 (1.79%) received a new diagnosis of TB at least 6 months after their RA diagnosis (Figure 1). Of the 518 patients with TB that we identified, the TB was pulmonary for 457 (88.22%) and extrapulmonary for 61 (11.78%). The incidence rate for TB was higher among RA patients than controls (3.4 vs. 1.4 cases/1,000 person-years, respectively; Table 2). After multivariable analysis and adjustment for age, sex, Charlson Comorbidity Index score, and urbanization, the risk for TB development increased 2.28-fold for RA patients over that for controls (95% CI 2.06–2.54; p<0.0001). The risk for TB development, after stratification for age, sex, and urbanization, was higher for RA patients than controls. Further analysis indicated that the risk for TB development was higher for RA patients with concurrent conditions than for controls with the same conditions.

**Table 2:** Subgroup analysis for new-onset TB and NTM among 144,985 patients with and without RA, Taiwan, 2001–2011*

Variable	With RA				Without RA			aHR (95% CI)‡	p value
	No. events	No. person-years	Incidence†		No. events	No. person-years	Incidence†		
New-onset TB									
All patients	518	150,284.0	3.4		1626	119,2456.6	1.4	2.28 (2.06–2.54)	<0.0001
Age at entry, y									
18–44	54	42,192.3	1.3		130	321,916.2	0.4	2.54 (1.78–3.63)	<0.0001
45–64	215	78,297.6	2.7		689	627,394.5	1.1	2.23 (1.90–2.63)	<0.0001
>65	249	29,794.0	8.4		807	243,145.9	3.3	2.28 (1.96–2.65)	<0.0001
Sex									
F	307	118,858.3	2.6		909	936,265.5	1.0	2.38 (2.08–2.74)	<0.0001
M	211	31,425.7	6.7		717	256,191.2	2.8	2.16 (1.84–2.54)	<0.0001
Concurrent condition									
CVD	204	41,104.2	5.0		671	270,893.6	2.5	1.99 (1.69–2.34)	<0.0001
DM	58	11,952.4	4.9		261	89,868.3	2.9	1.69 (1.26–2.27)	0.001
Hyperlipidemia	59	14,371.2	4.1		137	83,859.8	1.6	2.49 (1.80–3.43)	<0.0001
Liver cirrhosis	45	14,122.1	3.2		123	72,188.4	1.7	1.89 (1.33–2.69)	0.0001
COPD	74	11,232.7	6.6		343	77,702.4	4.4	1.40 (1.08–1.81)	0.011
Urbanization									
Urban	265	88,389.5	3.0		823	708,145.9	1.2	2.39 (2.06–2.77)	<0.0001
Suburban	80	21,371.3	3.7		257	169,798.3	1.5	2.16 (1.66–2.82)	<0.0001
Rural	173	40,523.2	4.3		546	314,512.5	1.7	2.20 (1.84–2.64)	<0.0001
New-onset NTM									
All patients	52	151,633.4	0.3		79	1,198,369.7	0.1	6.24 (4.24–9.17)	<0.0001
Age at entry, y									
18–44	6	42,367.8	0.1		12	322,534.9	0.0	3.70 (1.22–11.24)	<0.05
45–64	25	78,903.7	0.3		36	630,397.4	0.1	6.89 (3.93–12.07)	<0.0001
>65	21	30,361.9	0.7		31	245,437.5	0.1	6.71 (3.67–12.28)	<0.0001
Sex									
F	30	119,677.3	0.3		55	939,708.1	0.1	5.80 (3.55–9.47)	<0.0001
M	22	31,956.1	0.7		24	258,661.7	0.1	7.08 (3.77–13.29)	<0.0001
Concurrent condition									
CVD	15	41,555.1	0.4		24	273,076.0	0.1	5.03 (2.51–10.07)	<0.0001
DM	5	12,087.8	0.4		7	90,698.9	0.1	9.84 (2.79–34.75)	<0.001
Hyperlipidemia	4	14,523.6	0.3		6	84,326.4	0.1	6.16 (1.46–26.02)	<0.05
Liver cirrhosis	4	14,219.5	0.3		7	72,638.1	0.1	4.77 (1.24–18.38)	<0.05
COPD	5	11,451.8	0.4		15	78,904.5	0.2	3.02 (1.04–8.78)	<0.05
Urbanization									
Urban	27	89,070.6	0.3		38	711,281.4	0.1	5.91 (3.42–10.21)	<0.0001
Suburban	11	21,556.7	0.5		10	170,651.8	0.1	13.85 (5.32–36.03)	<0.0001
Rural	14	41,006.0	0.3		31	316,436.5	0.1	4.51 (2.28–8.93)	<0.0001

*aHR, HR, adjusted hazard ratio; COPD, chronic obstructive pulmonary disease; CVD, cardiovascular disease; DM, diabetes mellitus; NTM, nontuberculous mycobacteria; RA, rheumatoid arthritis TB, tuberculosis. †Rate per 1,000 person-years. ‡Adjusted for age, sex, Charlson Comorbidity Index score, and urbanization.

New-onset NTM disease developed in 131 (0.09%) patients (Figure 1). For 52 (0.18%) of the 28,997 RA patients, NTM disease was newly diagnosed at least 6 months after RA was diagnosed (Figure 1). Of these 52 patients with identified NTM, 27 (51.92%) had pulmonary and 25 (48.08%) had extrapulmonary disease; 27 (51.92%) had a history of previous TB. The incidence rate of NTM disease was significantly higher among RA patients than controls (0.3 vs. 0.1 cases/1,000 person-years, respectively; Table 2). Multivariable analysis indicated that risk for development of NTM disease was 6.24-fold higher among RA patients than controls (95% CI 4.24–9.17, p<0.0001). As was the case for TB, the risk for development of NTM disease after stratification for age, sex, and urbanization was higher for RA patients than controls. In addition, risk for development of NTM disease was higher for RA patients with concurrent conditions than for controls with the same conditions.

### Risk Factors for Mycobacterial Disease Development

**Figure 2:**
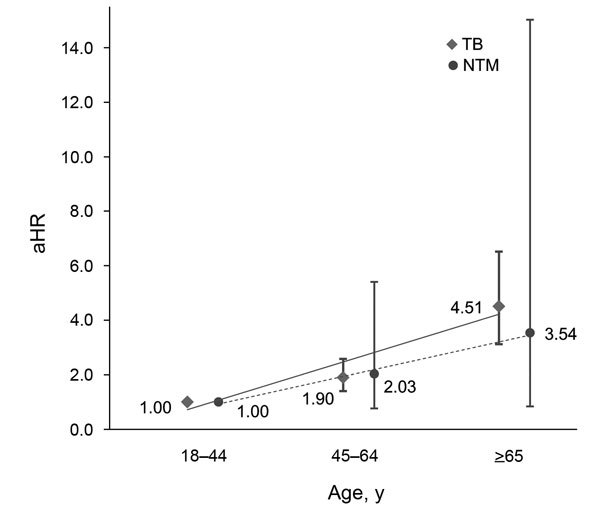
Adjusted hazard ratios (aHRs) and 95% CIs (error bars) for tuberculosis and nontuberculous mycobacteria infection according to age among patients with rheumatoid arthritis and matched controls. Increased risk correlated with increased age, Taiwan, 2001–2011.

Risk factors for development of mycobacterial diseases in RA patients were older age and male sex. For patient age >65 years, the adjusted hazard ratio [aHR] was 5.06 (95% CI 3.72–6.88; p<0.0001) for TB and 4.70 (95% CI 1.82–12.13; p = 0.001) for NTM. For male patients, aHR was 2.35 (95% CI 1.97–2.81; p<0.0001) for TB and 2.64 (95% CI 1.52–4.60; p = 0.001) for NTM (Table 3). In addition, among RA patients, the risk for TB and NTM was positively correlated with increased age (Figure 2). No significant difference in urbanization was found for TB and NTM disease development in RA patients.

**Table 3:** Multivariable analysis of baseline factors for 28,997 RA patients with new-onset TB and NTM, Taiwan, 2001–2011*

Variables	aHR†	95% CI	p value
TB			
Age at entry, y			
18–44	1.00	Reference	Reference
45–64	1.96	1.45–2.65	<0.0001
>65	5.06	3.72–6.88	<0.0001
Sex			
F	1.00	Reference	Reference
M	2.35	1.97–2.81	<0.0001
Charlson Comorbidity Index score			
0	1.00	Reference	Reference
1	1.08	0.87–1.34	0.513
2	1.14	0.89–1.46	0.305
>3	1.19	0.91–1.54	0.202
Urbanization			
Urban	1.00	Reference	Reference
Suburban	1.09	0.84–1.40	0.525
Rural	1.19	0.98–1.45	0.087
NTM			
Age at entry, y			
18–44	1.00	Reference	Reference
45–64	2.25	0.91–5.53	0.078
>65	4.70	1.82–12.13	0.001
Sex			
F	1.00	Reference	Reference
M	2.64	1.52–4.60	0.001
Charlson Comorbidity Index score			
0	1.00	Reference	Reference
1	1.64	0.85–3.19	0.143
2	1.31	0.59–2.94	0.509
>3	1.01	0.39–2.64	0.983
Urbanization			
Urban	1.00	Reference	Reference
Suburban	1.34	0.65–2.75	0.425
Rural	0.85	0.43–1.66	0.631

*aHR, adjusted hazard ratio; NTM, nontuberculous mycobacteria; RA, rheumatoid arthritis; TB, tuberculosis. †Adjusted for age, sex, Charlson Comorbidity Index score, and urbanization.

### Risk for Death among RA Patients with Mycobacterial Disease

Risk for death was higher among RA patients with mycobacterial infection than among RA patients without mycobacterial infection (aHR 1.62, 95% CI 1.38–1.90, p<0.0001 for TB; aHR 3.06, 95% CI 1.53–6.12, p = 0.002 for NTM; Table 4). Furthermore, the risk for death was increased among older RA patients (>65 years, aHR 8.81, 95% CI 7.48–10.39; p<0.0001), male patients (aHR 1.73, 95% CI 1.59–1.88; p<0.0001), and patients who lived in rural areas (aHR 1.41, 95% CI 1.29–1.54; p<0.0001).

**Table 4:** Multivariable analysis for risk for death among 28,997 RA patients with and without TB and NTM, Taiwan, 2001–2011*

Variable	aHR†	95% CI	p value
RA without TB or NTM	1.00	Reference	Reference
RA with TB	1.62	1.38–1.90	<0.0001
RA with NTM	3.06	1.53–6.12	0.002
RA with TB and NTM	1.58	0.82–3.04	0.174
Age at entry, y			
18–44	1.00	Reference	Reference
45–64	1.91	1.61–2.27	<0.0001
>65	8.81	7.48–10.39	<0.0001
Sex			
F	1.00	Reference	Reference
M	1.73	1.59–1.88	<0.0001
Charlson Comorbidity Index score			
0	1.00	Reference	Reference
1	1.05	0.95–1.17	0.357
2	1.25	1.12–1.41	0.0001
>3	1.92	1.73–2.14	<0.0001
Urbanization			
Urban	1.00	Reference	Reference
Suburban	1.27	1.13–1.42	<0.0001
Rural	1.41	1.29–1.54	<0.0001

*aHR, adjusted hazard ratio; NTM, nontuberculous mycobacteria; RA, rheumatoid arthritis; TB, tuberculosis. †Adjusted for age, sex, Charlson Comorbidity Index score, and urbanization.

## Discussion

This nationwide population-based retrospective cohort study indicated that among the ≈20 million enrollees in Taiwan’s NHIRD, the risk for development of TB was 2.28-fold greater and the risk for development of NTM disease 6.24-fold greater among RA patients than among controls. Among RA patients, significant risk factors for development of mycobacterial disease were older age (>65 years) and male sex. Furthermore, the risk for death was higher among RA patients with mycobacterial infection than among RA patients without mycobacterial infection.

The incidence of TB (53.0 cases/100,000 population in 2012) and mortality rate (2.7 cases 100,000 population in 2012) were higher in Taiwan than in other Asian (e.g., Japan, Korea) or western countries (*7*). In this study, the incidence rate for TB was significantly higher among RA patients than controls (3.4 vs. 1.4/1,000 person-years, respectively). A population-based study in Europe showed that the risk for development of TB was 4-fold higher among RA patients than among the general population (*26*). Although the incidence rate for TB for the general population declined over time, the incidence rate for TB among RA patients was still higher than that among controls (*26*).

Our findings that male sex and older age are major risk factors for mycobacterial infection among RA patients in Taiwan are consistent with local laboratory-based data (*6*) and findings of systematic reviews in Asia (*16*,*27*). However, in Japan, South Korea, and most western countries, most NTM disease patients were female (*16*,*28*). This discrepancy may be associated with lifestyle, environmental factors, and genetic factors. More studies are required to confirm this hypothesis. We also found a history of TB for 27 (51.92%) of NTM-infected RA patients and 43 (54.43%) of 79 controls, similar to results from eastern Asia (*27*). Furthermore, our results illustrated that risk for development of NTM disease was 6.24-fold higher among RA patients than controls and that the risk for death among RA patients with NTM disease was 3.06-fold higher than that for RA patients without NTM disease. A recent population-based study in Canada demonstrated a 2.07-fold increased risk for NTM disease among RA patients and a 1.81-fold increased risk for death among RA patients with NTM disease (*29*). The risk for exposure to mycobacteria closely correlated with the prevalence of mycobacterial diseases. The prevalence of mycobacterial diseases is higher in Taiwan (*5*,*7*), thus leading to an increased risk for infection (aHRs 6.24 vs. 2.07) and infection-related death (aHRs 3.06 vs. 1.81) among RA patients in Taiwan and Canada, respectively.

Among participants in this study, the risk for death among RA patients with NTM disease was higher than that among RA patients TB (aHRs 3.06 vs.1.62). This finding might reflect increased incidence of NTM disease in Taiwan, but awareness of NTM disease is not enough. In Taiwan, cases of TB should be reported to the public health administration, and control measures should be implemented immediately. However, cases of NTM disease do not have to be reported, and few hospitals can fully identify NTM (*6*). Furthermore, the severity of NTM disease varies, resulting in missed diagnoses for patients with mild and asymptomatic infection. Therefore, it is easy to ignore and underestimate the consequences of NTM disease. According to experience in Europe (*26*), risk for mycobacterial diseases in RA patients is reduced by increasing the awareness of hospital staff and screening for mycobacterial diseases. In addition, more laboratory data are needed for investigation of the characteristics (e.g., species distribution, invasion site, drug resistant) of clinical NTM strains.

In this study, pulmonary invasion affected most RA patients (88.22%) and controls (92.50%) with TB and slightly more than half (51.92%) of the RA patients with NTM disease. In the past few years, the incidence of NTM-associated pulmonary diseases and hospitalization has increased in Europe and the United States (*30*,*31*), and this increase is most likely associated with the global trend toward more persons being of older age, increased use of immunosuppressive medication, elevated prevalence of immune-modulating concurrent conditions, and use of advanced diagnostic techniques (*16*). In addition to pulmonary NTM disease, an increased percentage of extrapulmonary NTM disease was noted in RA patients than controls (48.08% vs. 34.18%). Similar elevated manifestations of extrapulmonary NTM (44%) were reported from another study, which found NTM disease in RA patients receiving anti–TNF-α therapy in the United States (*32*,*33*).

Our results showed that the risk for development of mycobacterial diseases was higher among RA patients with concurrent medical conditions than among controls with the same conditions. These findings were similar to those of a population-based study in Canada (*29*). Accumulating evidence indicates that risk factors for TB are diabetes mellitus, liver cirrhosis, and chronic obstructive pulmonary disease (*19*). However, the relationship between other RA-related conditions (e.g., hyperlipidemia) and mycobacterial diseases remains unclear. In addition to demographic and host factors, geographic factors are associated with the development of mycobacterial diseases (*19*,*34*). A population-based study in the United States found that TB-related deaths were strongly predominant among the general population living in urban areas, but no such meaningful difference was found for NTM disease–related deaths (*34*). Our results showed that after stratification of data for urbanization, the risk for both TB and NTM disease among all RA patients was higher than that for controls, but no significant difference with regard to urbanization and mycobacterial disease development in RA patients was found. Urbanization had fewer effects on increasing the risk for development of mycobacterial diseases in RA patients in Taiwan.

The NHIRD contains complete medical information regarding patients, including prescription details, diagnoses based on the ICD-9-CM codes, registration files, and original claims records for reimbursement. However, this administrative database has several limitations. First, the NHIRD does not contain detailed information of lifestyle factors (e.g., smoking, alcohol abuse, or substance abuse) or individual health status (e.g., body mass index, malnutrition, genetic factors) that are associated with mycobacterial infection and RA (*19*,*35*). Another limitation is that the NHIRD only contains medical claims data, so the current condition of the patients is unknown. Of note, the NHIRD does not include the results of laboratory examinations. To improve accuracy of the analysis, we used not only ICD-9-CM codes but also mycobacteria laboratory examination codes and antimycobacterial therapy receipts to identify TB and NTM disease cases. Because most patients with NTM pulmonary lung disease do not receive treatment, our definition (which requires treatment) is probably relatively insensitive, missing all untreated cases. The definition is undoubtedly highly specific, but because extrapulmonary NTM is almost always treated, our definition probably resulted in a higher than expected proportion of extrapulmonary NTM cases. The major strength of this study is use of a nationwide database with medical care records, which is minimally affected by selection and recall biases. In addition, the large sample size of the NHIRD (≈20 million enrollees, including patients and the general population) and long-term records enhanced the statistical power and accuracy of this study.

Increasing evidence indicates that the elevated risk for mycobacterial diseases among RA patients is primarily attributable to the effect of immunosuppressive therapies (*15*,*32*,*33*). Because most RA patients in Taiwan were treated with disease-modifying antirheumatic drugs or biologicals, we hypothesized that the risk for development of mycobacterial diseases may be associated with immunosuppressive therapy, RA-related concurrent conditions, or immune dysfunction caused by RA itself. Further studies are required to confirm this hypothesis. 

In conclusion, our results showed that risk for death is higher among RA patients with than without mycobacterial diseases. Therefore, TB- or NTM-infected RA patients should be closely monitored, especially those who are male, are >65 years of age, or have concurrent conditions.

## References

[1] World Health Organization G. Global tuberculosis report 2013 [cited 2014 Nov 15]. http://www.who.int/tb/publications/global_report/en/

[2] Falkinham JO. Impact of human activities on the ecology of nontuberculous mycobacteria. Future Microbiol. 2010;5:951–60 . 10.2217/fmb.10.53 20521938

[3] Griffith DE, Aksamit T, Brown-Elliott BA, Catanzaro A, Daley C, Gordin F, An official ATS/IDSA statement: diagnosis, treatment, and prevention of nontuberculous mycobacterial diseases. Am J Respir Crit Care Med. 2007;175:367–416. 10.1164/rccm.200604-571ST 17277290

[4] Thomson RM. Changing epidemiology of pulmonary nontuberculous mycobacteria infections. Emerg Infect Dis. 2010;16:1576–83. 10.3201/eid1610.091201 20875283 PMC3294381

[5] Brode SK, Daley CL, Marras TK. The epidemiologic relationship between tuberculosis and non-tuberculous mycobacterial disease: a systematic review. Int J Tuberc Lung Dis. 2014;18:1370–7. 10.5588/ijtld.14.0120 25299873

[6] Chien JY, Lai CC, Sheng WH, Yu CJ, Hsueh PR. Pulmonary infection and colonization with nontuberculous mycobacteria, Taiwan, 2000–2012. Emerg Infect Dis. 2014;20:1382–5. 10.3201/eid2008.131673 25062534 PMC4111185

[7] Centers for Disease Control. R.O.C. (Taiwan). Statistics of communicable diseases and surveillance report 2012. Taipei City (Taiwan): The Centers; 2013.

[8] Firestein GS. Evolving concepts of rheumatoid arthritis. Nature. 2003;423:356–61. 10.1038/nature01661 12748655

[9] Helmick CG, Felson DT, Lawrence RC, Gabriel S, Hirsch R, Kwoh CK, Estimates of the prevalence of arthritis and other rheumatic conditions in the United States. Part I. Arthritis Rheum. 2008;58:15–25. 10.1002/art.23177 18163481

[10] Lee J, Dunlop D, Ehrlich-Jones L, Semanik P, Song J, Manheim L, Public health impact of risk factors for physical inactivity in adults with rheumatoid arthritis. Arthritis Care Res (Hoboken). 2012;64:488–93. 10.1002/acr.21582 22278986 PMC3315605

[11] Doran MF, Crowson CS, Pond GR, O'Fallon WM, Gabriel SE. Frequency of infection in patients with rheumatoid arthritis compared with controls: a population-based study. Arthritis Rheum. 2002;46:2287–93. 10.1002/art.10524 12355475

[12] Carmona L, Hernandez-Garcia C, Vadillo C, Pato E, Balsa A, Gonzalez-Alvaro I, Increased risk of tuberculosis in patients with rheumatoid arthritis. J Rheumatol. 2003;30:1436–9 . 12858438

[13] Keane J. TNF-blocking agents and tuberculosis: new drugs illuminate an old topic. Rheumatology (Oxford). 2005;44:714–20. 10.1093/rheumatology/keh567 15741198

[14] Chen DY, Shen GH, Chen YM, Chen HH, Hsieh CW, Lan JL. Biphasic emergence of active tuberculosis in rheumatoid arthritis patients receiving TNFα inhibitors: the utility of IFNγ assay. Ann Rheum Dis. 2012;71:231–7. 10.1136/annrheumdis-2011-200489 22021896

[15] Winthrop KL, Baxter R, Liu L, Varley CD, Curtis JR, Baddley JW, Mycobacterial diseases and antitumour necrosis factor therapy in USA. Ann Rheum Dis. 2013;72:37–42. 10.1136/annrheumdis-2011-200690 22523429

[16] Kendall BA, Winthrop KL. Update on the epidemiology of pulmonary nontuberculous mycobacterial infections. Semin Respir Crit Care Med. 2013;34:87–94. 10.1055/s-0033-1333567 23460008

[17] Dougados M, Soubrier M, Antunez A, Balint P, Balsa A, Buch MH, Prevalence of comorbidities in rheumatoid arthritis and evaluation of their monitoring: results of an international, cross-sectional study (COMORA). Ann Rheum Dis. 2014;73:62–8. 10.1136/annrheumdis-2013-204223 24095940 PMC3888623

[18] Lindhardsen J, Ahlehoff O, Gislason GH, Madsen OR, Olesen JB, Torp-Pedersen C, The risk of myocardial infarction in rheumatoid arthritis and diabetes mellitus: a Danish nationwide cohort study. Ann Rheum Dis. 2011;70:929–34 . 10.1136/ard.2010.143396 21389043

[19] Marais BJ, Lonnroth K, Lawn SD, Migliori GB, Mwaba P, Glaziou P, Tuberculosis comorbidity with communicable and non-communicable diseases: integrating health services and control efforts. Lancet Infect Dis. 2013;13:436–48. 10.1016/S1473-3099(13)70015-X 23531392

[20] Lee CH, Lee MC, Lin HH, Shu CC, Wang JY, Lee LN, Pulmonary tuberculosis and delay in anti-tuberculous treatment are important risk factors for chronic obstructive pulmonary disease. PLoS ONE. 2012;7:e37978. 10.1371/journal.pone.0037978 22662259 PMC3360660

[21] Lee CH, Lee MC, Shu CC, Lim CS, Wang JY, Lee LN, Risk factors for pulmonary tuberculosis in patients with chronic obstructive airway disease in Taiwan: a nationwide cohort study. BMC Infect Dis. 2013;13:194. 10.1186/1471-2334-13-194 23631563 PMC3652752

[22] Yeh JJ, Wang YC, Lin CL, Chou CY, Yeh TC, Wu BT, Nontuberculous mycobacterial infection is associated with increased respiratory failure: a nationwide cohort study. PLoS ONE. 2014;9:e99260. 10.1371/journal.pone.0099260 24918925 PMC4053398

[23] The National Health Insurance Statistics. Statistics & surveys [cited 2014 Nov 15]. http://www.nhi.gov.tw/English/webdata/webdata.aspx?menu=11&menu_id=296&webdata_id=1942&WD_ID=296

[24] Arnett FC, Edworthy SM, Bloch DA, McShane DJ, Fries JF, Cooper NS, The American Rheumatism Association 1987 revised criteria for the classification of rheumatoid arthritis. Arthritis Rheum. 1988;31:315–24. 10.1002/art.1780310302 3358796

[25] Quan H, Sundararajan V, Halfon P, Fong A, Burnand B, Luthi JC, Coding algorithms for defining comorbidities in ICD-9-CM and ICD-10 administrative data. Med Care. 2005;43:1130–9. 10.1097/01.mlr.0000182534.19832.83 16224307

[26] Arkema EV, Jonsson J, Baecklund E, Bruchfeld J, Feltelius N, Askling J; ARTIS Study Group. Are patients with rheumatoid arthritis still at an increased risk of tuberculosis and what is the role of biological treatments? Ann Rheum Dis. 2015;74:1212–7 . 10.1136/annrheumdis-2013-204960 24608401

[27] Simons S, van Ingen J, Hsueh PR, Van Hung N, Dekhuijzen PN, Boeree MJ, Nontuberculous mycobacteria in respiratory tract infections, eastern Asia. Emerg Infect Dis. 2011;17:343–9. 10.3201/eid170310060 21392422 PMC3165997

[28] Morimoto K, Iwai K, Uchimura K, Okumura M, Yoshiyama T, Yoshimori K, A steady increase in nontuberculous mycobacteriosis mortality and estimated prevalence in Japan. Ann Am Thorac Soc. 2014;11:1–8. 10.1513/AnnalsATS.201303-067OC 24102151

[29] Brode SK, Jamieson FB, Ng R, Campitelli MA, Kwong JC, Paterson JM, Risk of mycobacterial infections associated with rheumatoid arthritis in Ontario, Canada. Chest. 2014;146:563–72. 10.1378/chest.13-2058 24384637

[30] Ringshausen FC, Apel RM, Bange FC, de Roux A, Pletz MW, Rademacher J, Burden and trends of hospitalisations associated with pulmonary non-tuberculous mycobacterial infections in Germany, 2005–2011. BMC Infect Dis. 2013;13:231. 10.1186/1471-2334-13-231 23692867 PMC3667050

[31] Billinger ME, Olivier KN, Viboud C, de Oca RM, Steiner C, Holland SM, Nontuberculous mycobacteria–associated lung disease in hospitalized persons, United States, 1998–2005. Emerg Infect Dis. 2009;15:1562–9. 10.3201/eid1510.090196 19861046 PMC2866394

[32] Winthrop KL, Chang E, Yamashita S, Iademarco MF, LoBue PA. Nontuberculous mycobacteria infections and anti–tumor necrosis factor-α therapy. Emerg Infect Dis. 2009;15:1556–61. 10.3201/eid1510.090310 19861045 PMC2866401

[33] Winthrop KL, Iseman M. Bedfellows: mycobacteria and rheumatoid arthritis in the era of biologic therapy. Nat Rev Rheumatol. 2013;9:524–31. 10.1038/nrrheum.2013.82 23797309

[34] Mirsaeidi M, Machado RF, Garcia JG, Schraufnagel DE. Nontuberculous mycobacterial disease mortality in the United States, 1999–2010: a population-based comparative study. PLoS ONE. 2014;9:e91879. 10.1371/journal.pone.0091879 24632814 PMC3954860

[35] Symmons DP, Bankhead CR, Harrison BJ, Brennan P, Barrett EM, Scott DG, Blood transfusion, smoking, and obesity as risk factors for the development of rheumatoid arthritis: results from a primary care–based incident case–control study in Norfolk, England. Arthritis Rheum. 1997;40:1955–61. 10.1002/art.1780401106 9365083

